# NAViGaTing the Micronome – Using Multiple MicroRNA Prediction Databases to Identify Signalling Pathway-Associated MicroRNAs

**DOI:** 10.1371/journal.pone.0017429

**Published:** 2011-02-25

**Authors:** Elize A. Shirdel, Wing Xie, Tak W. Mak, Igor Jurisica

**Affiliations:** 1 Department of Medical Biophysics, University of Toronto, Toronto, Ontario; 2 Ontario Cancer Institute, Princess Margaret Hospital/University Health Network and The Campbell Family Institute for Cancer Research, Toronto, Ontario, Canada; 3 Campbell Family Institute for Breast Cancer Research, Ontario Cancer Institute, Princess Margaret Hospital/University Health Network, Toronto, Ontario, Canada; 4 Department of Computer Science, University of Toronto, Toronto, Ontario, Canada; Bellvitge Biomedical Research Institute (IDIBELL), Spain

## Abstract

**Background:**

MicroRNAs are a class of small RNAs known to regulate gene expression at the transcript level, the protein level, or both. Since microRNA binding is sequence-based but possibly structure-specific, work in this area has resulted in multiple databases storing predicted microRNA:target relationships computed using diverse algorithms. We integrate prediction databases, compare predictions to *in vitro* data, and use cross-database predictions to model the microRNA:transcript interactome – referred to as the *micronome* – to study microRNA involvement in well-known signalling pathways as well as associations with disease. We make this data freely available with a flexible user interface as our microRNA Data Integration Portal — mirDIP (http://ophid.utoronto.ca/mirDIP).

**Results:**

mirDIP integrates prediction databases to elucidate accurate microRNA:target relationships. Using NAViGaTOR to produce interaction networks implicating microRNAs in literature-based, KEGG-based and Reactome-based pathways, we find these signalling pathway networks have significantly more microRNA involvement compared to chance (p<0.05), suggesting microRNAs co-target many genes in a given pathway. Further examination of the micronome shows two distinct classes of microRNAs; *universe microRNAs*, which are involved in many signalling pathways; and *intra-pathway microRNAs*, which target multiple genes within one signalling pathway. We find universe microRNAs to have more targets (p<0.0001), to be more studied (p<0.0002), and to have higher degree in the KEGG cancer pathway (p<0.0001), compared to intra-pathway microRNAs.

**Conclusions:**

Our pathway-based analysis of mirDIP data suggests microRNAs are involved in intra-pathway signalling. We identify two distinct classes of microRNAs, suggesting a hierarchical organization of microRNAs co-targeting genes both within and between pathways, and implying differential involvement of universe and intra-pathway microRNAs at the disease level.

## Introduction

MicroRNAs are short, but important non-coding RNA sequences that regulate gene expression [1]. They are thought to target the 3′ Untranslated Regions (UTRs) of mRNA, disrupting their ability to be translated into proteins, sometimes repressing the expression of the mRNA itself [2], [3], [4], [5], [6], [7], [8], [9]. MicroRNA prediction algorithms generally pair the seed region of the microRNA (bases 2–8 from the 5′ end of the microRNA) to a cognate mRNA sequence. However, this binding is complicated by many factors, not the least of which is that imperfect microRNA:mRNA binding occurs, and thus single base-pair mismatches and G:U wobble base-pairs must be considered.

Discovery of the first microRNA – lin-4 in worm (*C. Elegans*) [10], its further characterization in 1989 [11], annotation as a non-coding RNA in 1993 with a sequence complementary to the lin-14 3′ UTR [1], [12], and functional characterization as having a translational repression effect later that year [13] opened a rich research field. Many subsequent *in vitro* experiments and computational predictions aimed at uncovering microRNA:target relationships to fathom microRNA effects on gene expression regulation. With the discovery of a second nematode microRNA – let-7, which targets lin-41 and hbl-1, the concept of microRNAs made the jump from worms to higher species, since let-7 had well-known homologues even in humans [14], [15], [16]. Coining the term “microRNA” for this class of non-coding gene regulators in three back-to-back Science papers in 2001 [17], [18], [19], the discovery of microRNAs had crossed over to the human domain, and finding microRNA targets became a high priority. After the first bioinformatics attempt at predicting plant microRNAs [5], many microRNA prediction algorithms, for both fly (*D. melanogaster*) and human (*H. sapiens*), were developed [20], [21], [22]. More than 10 public databases for microRNA:mRNA target prediction have been created, all using different algorithms and approaches. Considering varying degrees of sequence similarity, conservation, site accessibility and different targeted regions of the mRNA – all databases add a novel level of complexity to the microRNA question [20], [23], [24], [25], [26], [27], [28], [29], [30], [31], [32], [33], [34], [35], [36], [37], [38], [39], [40].

To visualize and analyze these complex relationships between different predictions of microRNA:mRNA target mappings, we borrow ideas from protein-protein interactions and gene regulatory networks. We first integrate all databases into a freely available data portal – mirDIP (microRNA Data Integration Portal) – and use NAViGaTOR (Network Analysis, Visualization and Graphing Toronto) [41] to analyze and visualize the resulting network of microRNA:mRNA target mappings – the microRNA interaction network (*micronome)*.

## Results and Discussion

### Characteristics of microRNA predicting databases

There are many characteristics of microRNA:mRNA target binding that are taken into account - in different combinations - for each microRNA prediction database. We begin with a review of these criteria. Table 1 shows all databases considered in this research. To enable more informed integration of these predictions, we consider characteristics of individual microRNA prediction algorithms in detail, and summarize them in Table 2. All eleven main groups of features used for prediction are described below:

1. *Seed Sequence match.* All prediction algorithms depend on this criterion. Allowing for base-pair mismatches and G:U wobbles, which have been shown to be important in microRNA binding [42], prediction algorithms look for high degree of complementarity between the 5′ end of the microRNA and the 3′ end of the mRNA target sequence. Particular attention is paid to the seed region (bases 2–8 from the microRNA 5′ end).
2. *Conservation.* Many prediction algorithms take into account the conservation of the microRNA binding sequence in the mRNA target. Generally used as a filtering step, a highly conserved target site is thought to produce a more reliable prediction. Conservation is not directly used in some databases (Probability of Interaction by Target Accessibility (PITA)) [30], [34], is not directly incorporated into the score in others (Targetscan) [24], [27], [33], and is not used at all in others (RNA22) [34]. Interestingly, PITA results suggest that considering site accessibility is analogous to considering conservation, since accessible 3′ UTR microRNA binding sites tend to fall in conserved regions [30]. To reduce bias in our analyses, we use both predictions with and without conservation.
3. *Free Energy of microRNA:mRNA duplex.* The Free Energy of the microRNA:mRNA duplex (ΔG), is often calculated with the Vienna Folding package [43], [44], [45] or RNA hybrid [46]. It evaluates the energy required for the formation of the microRNA:mRNA duplex from a completely dissociated state – a more negative value indicates a larger inclination for the two RNAs to bind.
4. *Site accessibility.* Site accessibility is not considered in many prediction algorithms. Measured as ΔΔG for use in PITA, it compares the energy requirement for the already folded 3′ UTR to unfold to allow the microRNA accessibility to the target site, and to refold into the microRNA:mRNA duplex [30]. A more negative ΔΔG indicates a favourable folding energy for the microRNA:mRNA configuration.
5. *Contribution of multiple binding sites.* Many algorithms reward microRNAs that have multiple binding sites within the 3′ UTR of a particular gene, reasoning that the microRNAs will be able to exert a dose-dependent effect on target expression. Binding sites can be for a single microRNA or for multiple different microRNAs that show co-operativity resulting in synergistic gene repression [47]. Several studies have shown that the ideal inter-binding site distance falls between 8–40 base-pairs [27], [48].
6. *Local ALU content.* ALU sequences are segments of repetitive DNA interspersed within the human genome, thought to have arisen through retro-transposons and so named because they can be cleaved by the restriction enzyme Alu1 (reviewed in [49]). Considered in Targetscan's context score, Grimson et al. have shown that an enrichment of A or U base-pairs in the 30 nucleotides up or downstream of the microRNA binding site in the 3′ UTR tends to favorably associate with repression in target expression [27], [33].
7. *Local mRNA sequence.* The consideration of sequence surrounding the microRNA binding site on the 3′ UTR is sometimes taken into account. Algorithms may examine local sequence effect on site accessibility, or examine sequence content for particular nucleotides [27], [30], [33].
8. *Ribosomal shadow.* Considered in Targetscan, the 15 nucleotides after the stop codon in a 3′ UTR form poor microRNA target binding sites that show little ability to repress expression. It has been postulated that this is due to a ribosomal shadow effect [27].
9. *Uses miRanda.* miRanda [20], [29] is the first microRNA alignment algorithm, is similar to the Smith-Waterman algorithm for sequence alignment and uses rules of thumb previously established in sequence alignment [50], [51], [52]. It forms the basis of several microRNA prediction algorithms. miRanda considers several features described below:
  - Sequence match – a reward of +5 for a G = C or A = U match, +2 for G:U wobble. A penalty of −2 for a Gap Extension and −8 for a Gap Opening. The cutoff for S, the result of these sequence matches is generally S>80 (flies), S>50 (humans).
  - Scaling – Matches in positions 1–11 of the microRNA (from the 5′ end) are given twice the weight of matches elsewhere to reflect the asymmetry of microRNA binding [29].
  - Four empirical rules:
    - No mismatch in bases 2–4;
    - <5 mismatches in bases 3–12;
    - At least 1 mismatch in bases 9 to (Length-5);
    - <2 mismatches in the final 5 base-pairs.
  - Vienna Package Folding assumes the microRNA is linked to the 3′ UTR by 8 –x– base-pairs that cannot bind anything. This single structure is then folded. The ΔG cutoff is usually set as ΔG<−14 kcal/mol for flies and ΔG<−17 kcal/mol for humans.
  The final score is the total energy and total score of all hits between those of a microRNA and a 3′ UTR.
  - Conservation – a filtering step requiring 90% conservation or more between human and rat or mouse and 80% conservation between *D. melanogaster* and *D. pseudoobscura* or *A. gambiae.*
10. *Position effects.* Positional effects reward microRNA target sites that fall within the first quartile of the 3′ UTR after the stop codon (+15 base-pairs) or within the final quartile of the 3′ UTR, near the poly(AAAA) tail. This effect is more pronounced in long UTRs [27].
11. *3′ Pairing.* Aside from strong seed region pairing, many algorithms that aren't based on miRanda also require nucleotide binding between the microRNA and the target mRNA between bases 12–17 of the 3′ end of the microRNA [27].

**Table 1:** MicroRNA Prediction Databases.

Database	Details	MappedInteractions	MicroRNAs	UniqueMappedTargets
Targetscan	Conservation	189,075	675	16,512
Targetscan	No Cons.	1,457,484	677	17,678
RNA22	3′ UTR	264,630	313	14,949
RNA22	5′ UTR	53,405	313	7,333
RNA22	CDS	487,110	313	19,766
Microrna.org	Conservation	956,664	677	16,875
mirBase	Conservation	568,099	711	21,111
PITA	Top Hits	208,937	677	10,143
PITA*	All Hits	4,010,548	677	16,942
PicTar*	4-way	56,229	178	6,792
PicTar	5-way	17,224	129	2,534
microT$	v3.0	1,434,406	555	17,585

*Not used in all comparisons, nor the construction of microRNA interaction networks since it is a superset of the top database predictions.

$ Not used in all comparisons, nor the construction of microRNA interaction networks since it was not available for bulk download at the time of data curation.

**Table 2:** Characteristics of MicroRNA Prediction Databases.

	TargetscanConserved	TargetscanNon-Conserved	RNA22 3′ UTR	RNA22 5′ UTR	RNA22 CDS	microRNA.org	microCosm (formerly mirBase)	PITATop Hits	PITAAll Hits	picTar 4-way	picTar 5-way	DIANA microT
Conservation	X*					X	X	§	§	X	X	X
Site Accessibility								X	X			
Local AU content	X	X										
Multiple Binding Sites(1 microRNA)	X	X				X	X	X	X	X	X	X
Multiple Binding Sites(>1 microRNA)										X¥	X¥	
Uses miRanda						X	X					
Free Energy of Duplex						X	X	X	X	X	X	X
Examines surrounding Sequence	X	X						X	X			
Weighted 5′ end or considers seed type	X	X				X	X					X

*Targetscan Conserved uses conservation, but it is not integrated into the context score.

§ PITA does not explicitly use conservation in scoring targets. However, accessible microRNA binding sites tend to show high conservation.

¥ picTar does have predictions for multiple microRNAs binding to a single 3′ UTR; however, that data was not used in this study.

### MicroRNA prediction database similarities

**Figure 1:**
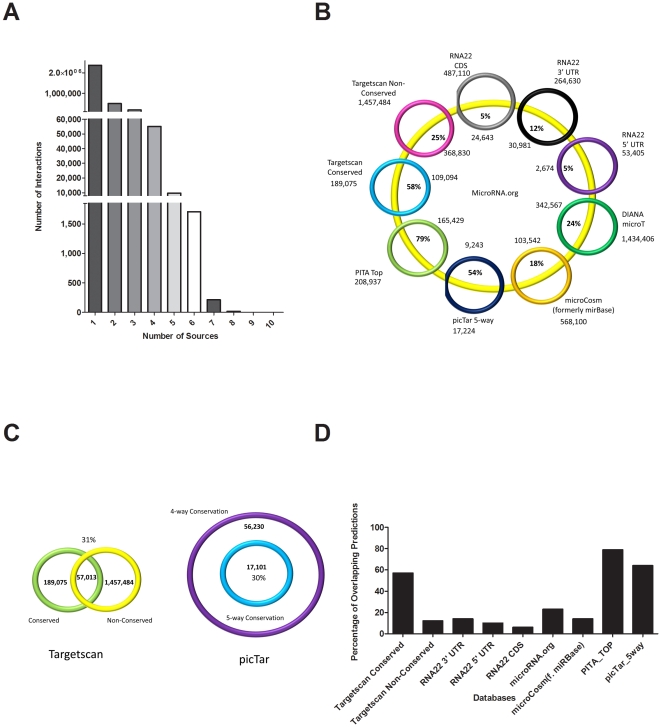
MicroRNA prediction database characteristics. Panel A: Distribution of microRNA:target predictions by number of predicting databases. Panel B: Overlap of microRNA prediction databases with microRNA.org. Panel C: Overlap of microRNA prediction databases Targetscan and picTar, since both consider degree of conservation as part of their scoring scheme. Panel D: Percentage of overlapping microRNA predictions across two or more databases.

Since microRNA:mRNA target prediction algorithms use different combinations of features to perform the same task, it is useful to analyze the distribution of these predictions across databases. There is an expected trend – with far fewer predictions being made that transcend six or more databases than those that are present in just one database. We count over 2 million predictions present in only one database, falling off to a surprisingly small 18 predictions identified in 8 of the 9 databases considered (As indicated in Table 1, we do not consider PITA All Targets nor picTar 4-way in this part of our analysis to avoid double-counting. Nor do we consider microT, since bulk download was not available at the time of data curation) (Figure 1A). Figure 1B compares all database predictions to microRNA.org – indicating that although we see low total overlap among all databases, in reference to the largest conservation-considering database there is considerable similarity between at least five database prediction schemes. Although DIANA microT v3.0 [39], [40] was not included in our extended database analysis and comparison, since it was not available for bulk download when our study began, we have included it in this figure for the sake of comparison.

Although most databases consider conservation, they each handle it differently. Bartel's Targetscan publishes dual lists of targets based on either conserved or non-conserved sites. Thirty-one percent of these microRNA:mRNA target predictions are shared by both lists (Figure 1C, left panel), demonstrating that there is a strong tendency for genes to contain both conserved and non-conserved microRNA binding sites along the length of their 3′ UTR. On the other hand, picTar considers grades of conservation in their prediction algorithm. Publishing both a 4-way and 5-way conservation scheme (human, mouse, rat, dog vs. human, mouse, rat, dog, chicken) picTar suggests degree of conservation correlates with robustness of prediction. In this case we can see that one list is clearly a subset of the other, and moving from a less conserved setting to a more conserved setting reduced the number of predicted targets by 30% (Figure 1C, right panel). When combining datasets, Figure 1D shows the percentage of predictions preserved per prediction scheme when requiring a microRNA:mRNA target prediction to occur in at least three databases. Targetscan and PITA Top Hits have the most remaining interactions after applying this filter.

### Comparing microRNA prediction databases to the truth

#### MicroRNA target filtering is vital

**Figure 2:**
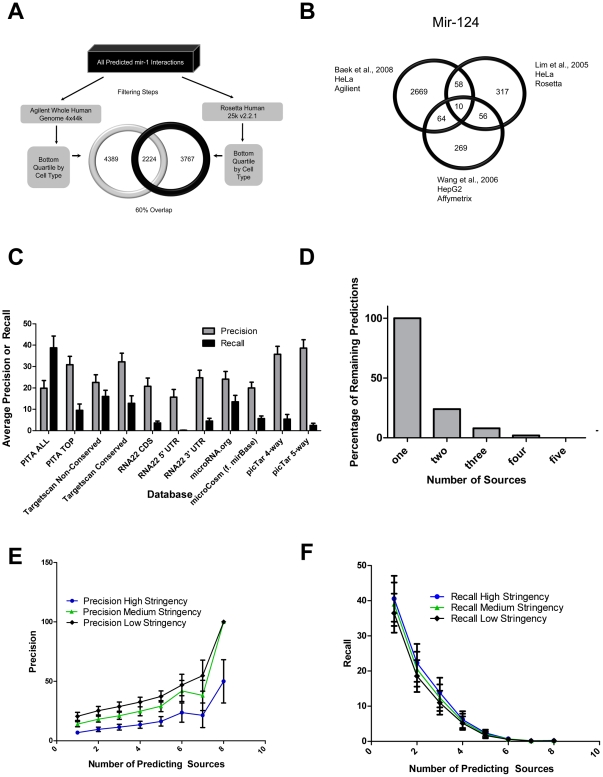
MicroRNA target prediction processing and evaluation. Panel A: Identification of microRNA targets is highly dependent on the experimental setup. Filtering by cell type and microarray platform on an identical initial prediction set can cause a divergence of up to 40% in the final target lists. Panel B: MicroRNA over-expression in different experimental settings results in poor overlap of identified targets. Venn diagram of discrepancy between *in vitro* microRNA over-expression experiments of mir-124. Panel C: Comparison of precision and recall across microRNA prediction databases, measured by computing the average values for all microRNA predictions by a particular database compared to their matched low stringency “ground truths”. Panel D: The percentage of remaining predictions by considering overlap across 2, 3, 4, and 5 prediction databases. Panel E: Precision measurements for microRNA:target predictions by number of prediction databases, indicating the percentage of predicted targets that were shown to be true across *in vitro* experiments. Stringency levels refer to confidence in the microarray data and were determined by either p-value or percentage knockdown as discussed in the methods. Panel F: Recall measurements for microRNA:target predictions by number of prediction databases, indicating the percentage of *in vitro* targets covered by predictions. Stringency levels refer to confidence in the microarray data and were determined by either p-value or percentage knockdown as discussed in the methods.

To examine whether a combination of microRNA prediction databases would outperform any one source, data from 15 publicly available microRNA over-expression/knockdown experiments followed by microarray [53], [54], [55], [56], [57], [58], [59], [60], [61] was assembled (Table 3). As discussed in the *Methods* section, when comparing microRNA target predictions to actual microRNA targets (as determined by microarray experiments) two filtering steps were performed to increase the suitability of the target predictions for the data – filtering by both microarray and by cell type. Filtering by microarray (Table 3 column 3) eliminates targets not present on the particular chip in the experiment, and thus having no chance of appearing in the final target set. Filtering by cell type (Table 3 column 4) eliminates genes expressed at only low levels in the cell line (which would reduce their chances of showing a knock-down effect). This two-step filtering drastically changes the predictions. As illustrated in Figure 2A, beginning with an identical set of mir-1 predicted targets across all databases and filtering by cell type and chip type to make the target predictions suitable for comparison to 2 different experiments results in significantly different final prediction sets – with overlapping targets numbering only 60% of the sets – clearly demonstrating the need to tailor predictions to the setting in which the experiment was done before any comparisons are undertaken. This filtering exercise shows how critical it is to consider tissue specificity when examining microRNAs of interest. Clearly, with the availability of more *in vitro* and *in vivo* data, it will become crucial to ensure that data is organized in a tissue-specific manner to enable more accurate modelling of the interactions present in particular settings.

**Table 3:** Characteristics of High-throughput Experiments.

Study	microRNA	Platform	Cell Type
Lim et al., 2005	hsa-mir-1	Rosetta 25 k v2.2.1	HeLa
Baek et al., 2008	hsa-mir-1	Agilent Whole Genome 4×44 k	HeLa
Linsley et al., 2007	hsa-mir-106b	Rosetta/Merck 44 k 1.1	HeLa
Lim et al., 2005	hsa-mir-124	Rosetta 25 k v2.2.1	HeLa
Wang et al., 2006	hsa-mir-124	Affymetrix U133 plus2	HepG2
Baek et al., 2008	hsa-mir-124	Agilent Whole Genome 4×44 k	HeLa
Ceppi et al., 2009	hsa-mir-155	Affymetrix U133 plus2	MDDS
Linsley et al., 2007	hsa-mir-16	Rosetta/Merck 44 k 1.1	HeLa
Baek et al., 2008	hsa-mir-181	Agilent Whole Genome 4×44 k	HeLa
Gennarino et al., 2009	hsa-mir-26b	Affymetrix U133 2	HeLa
Tavazoie et al., 2008	hsa-mir-335	Affymetrix U133 plus2	LM2
Huang et al., 2008	hsa-mir-373	Wistar Illumina V6	MCF-7
Huang et al., 2008	hsa-mir-520c	Wistar Illumina V6	MCF-7
Webster et al., 2009	hsa-mir-7	Affymetrix U133 plus2	A549
Gennarino et al., 2009	hsa-mir-98	Affymetrix U133 2	HeLa

#### High-throughput target validation experiments are not always in agreement

Ideally, high-throughput experiments would provide clear and concise answers in simple over-expression experiments. Unfortunately, we have not found that to be entirely the case. Examining the filtered results for the 2 microRNAs with high-throughput experimental results by multiple groups – there is remarkably little overlap between reported targets. Using mir-124 over-expression as an example, comparing the Baek et al. [53], Lim et al. [57] and Wang et al. [60] data sets – at the least stringent confidence level for targets, allowing the overlap between experiments to be maximal – we see only 10 common targets between all 3 lists, 3.7% of the smallest target list (Figure 2B). Expanding the overlap to include “true” targets predicted on 2 *in vitro* lists improves the situation, yet covers less than 50% of the smallest dataset. Similar results are seen in duplicate mir-1 experiments putting the overlap at 8%. One possible explanation for such observations is over-dosing with transfections, resulting in deregulation of gene expression due to a massive influx of microRNA molecules [62].

#### Comparing predictions to ground truth

PITA Top Targets, picTar 5-way Conservation and TargetScan Conserved Targets are all suitable candidates for top microRNA prediction database. Not only do they retain many predictions passing through a filter requiring predictions to be present in 3 or more databases (79%, 64%, 57% respectively) (Figure 1D), they also perform well when evaluating database performance on both precision and recall when compared to publicly available high-throughput microarray data (Figure 2C). While all three databases have many retained cross-database predicted targets, PITA and Targetscan Conserved do tend to outperform picTar 5-way when both precision and recall are considered – that is, when we require database prediction sets to not only contain many true positives, but also to predict many of the actual true targets. Examining the least stringent *in vitro* “ground truth” data: PITA Top Targets, picTar 5-way Conservation and Targetscan Conserved have precision and recall values of: 30%, 9%; 38%, 2%; 32%, 12% respectively. This demonstrates that although many of picTar 5-way's predictions are true, it performs exceptionally poorly when measuring the number of real targets that picTar actually predicts.

In the balance between precision and recall one might suggest using these databases as follows: 1) when looking for confirmatory evidence of a particular interaction between a microRNA and a specific target – it is better to use a database with superior recall such as Targetscan Conserved, Targetscan Non-Conserved or microCosm (formerly mirBase), which are more likely to include a target prediction if one exists; 2) when identifying any possible target for a particular microRNA to form the basis for *in vitro* or *in vivo* experiments, it would be best to consult picTar 5-way; 3) when finding *in silico* evidence for an interaction of a microRNA and a gene of a certain family or function, it is best to use a database with a more even balance between precision and recall such as PITA Top Targets.

#### Comparing predictions to Tarbase

Tarbase [63], curated by the DIANA Lab, provides a running list of microRNA interactions that have been shown to be true or false by either microarray experiments, pSILAC experiments or some other manner of specific probing for a particular microRNA:target interaction. Although Tarbase does not represent a non-biased list of microRNA targets, it is interesting to compare our list of 2+DB microRNA interactions to those present in their database. Thirty-nine percent of Tarbase-reported True mRNA repression targets, 48% of Tarbase-reported True mRNA cleavage targets, 67% of Tarbase-reported targets of unknown effect, 32% of Tarbase-reported pSILAC tested interactions and 62% of Tarbase-reported microarray tested interactions were present in our 2+DB set of interactions.

Since microRNAs act through translational inhibition more frequently than they do through mRNA degradation, it is obvious that examining microarray data is not the perfect setting in which to evaluate microRNA targets. The subset of targets that have been transcribed but not translated will still be expressed in the data and as such they will be missed. However, it has been shown that proteins repressed by more than 30% also tend to destabilize at the transcript level [53] – meaning that examination of expression levels is a reasonable surrogate for large translational repressions. Another possible source for incorrect predictions includes off-target effects. MicroRNA overexpression is thought to produce some false positives, perhaps due to dosage issues [62]. However, these off-target effects will occur less frequently than in synthetic siRNA overexpression systems.

High-throughput proteomics approaches such as pSILAC experiments are exciting new techniques that are emerging at the forefront of microRNA target research, and which allow the direct comparison of the proteomes of two different samples. Although an improvement on expression analyses for microRNA target research, examinations at the protein level will still suffer from the inability to distinguish primary from secondary effects. Furthermore, they are neither as high-throughput as expression analyses nor as time-efficient to run, and the set-up costs to run mass spectrometry experiments are far higher than microarrays at the present time. Optimal microRNA target analysis would require experiments where it can be shown that actual microRNA:mRNA binding is occurring with an associated reduction in mRNA or protein expression. Only then could we be certain an interaction is occurring – and such high-throughput experiment series remain a future challenge.

#### Integrating prediction databases in mirDIP

Due to the massive amount of genomic information being deciphered on a daily basis, there is an inevitable bottleneck between computational prediction and identification of binding sites, and the *in vitro* or *in vivo* validation of such interactions. Clearly, it would be useful to be able to prioritize microRNA:mRNA target predictions to reduce excessive false leads and unnecessary experiments. It has been previously shown, and confirmed here that none of the microRNA prediction databases does a perfect job of target identification [53], [64], although they are all suitable to provide an initial prediction. Integrating multiple databases improves accuracy or coverage of predictions by balancing out the precision and recall. Comparing microRNA predictions made by a minimum of either two or three databases to all truth files, enables us to retain 24% and 8% of filtered target predictions (Figure 2D), and obtain precision and recall values of 25%, 19%; 29%, 11% respectively (Figures 2E, 2F), providing a more balanced precision:recall ratio.

To enable this analysis, we introduce mirDIP – the microRNA Data Integration Portal – a free and publicly available data portal integrating up-to-date microRNA target predictions from eleven individual source prediction databases [20], [23]–[35]. Similar to our Interologous Interaction Database (I2D) maintenance program, we will update it at minimum twice a year to ensure that the latest microRNA:target prediction data from all sources is available to users. Importantly, to ensure consistency and enable accurate re-analysis in the future using new and older data, we keep track of versions of individual resources, and all mirDIP releases will be able to search the most current, or older versions.

Similar to mirGator, which amalgamates three microRNA databases (miRanda, picTar and TargetScan) with expression data while also providing enrichment analysis [65], mirDIP allows the user to take more control over the prediction data that they consider. Not only does our resource conveniently integrate eleven different prediction databases in one place, it allows users to choose which combinations of databases they would like to consider – refining options by database or by database characteristics – when selecting prediction data. This empowers users to capitalize on their knowledge of the workings of different databases, compensating for strengths and weaknesses of individual databases – choosing to focus on schemes considering different variables to create a customized prediction set based on the user's preferences and tailored to application-specific tasks, taking into account the need for either high precision or high recall as discussed above. File S1 introduces the mirDIP interface (Figure S1) and describes several search scenarios. Figures S2, S3, S4, S5 display screenshots of the mirDIP search parameters. Finally, in the sections that follow, we describe how mirDIP can be used along with NAViGaTOR [41] – a scalable, network analysis and visualization system – to perform novel microRNA:target prediction visualization.

### Construction of microRNA interaction networks

For the construction of microRNA interaction networks based on gene signalling pathways, we have refrained from using only targets from *in vitro* or *in vivo* experiments due to the obvious bias present in such data. Rather, we have chosen to use interactions appearing at two different confidence levels – those present in at least 2, or at least 3 microRNA prediction databases (2+DB, 3+DB) as a threshold for robust microRNA:target predictions. Further, drawing from nine of the twelve databases indicated in Table 1 to determine the 2+DB and 3+DB datasets (eliminating the risk of double-counting by omitting the PITA All Targets and picTar 4-way databases and not including microT), we draw from 4/9 databases using conservation as a target site algorithm criterion and 5/9 databases not considering it. As such, we ensure that the requirement of sequence conservation does not influence the construction of microRNA networks in either direction.

Beginning with the well-known Phosphoinositide 3-Kinase (PI3K) pathway, we examined two aspects of this pathway with respect to microRNA involvement, garnering our pathway information from reviews discussing member-genes [66], [67], [68], [69].

#### PI3K subunit regulation

To examine the relevance of mapping microRNAs into signalling pathways, we chose to examine 2 separate coordinate signalling scenarios in the PI3K pathway. Well known for its control of a broad range of down-stream effector genes, the PI3K pathway is involved in cell growth, proliferation, differentiation, cell death, motility and survival. Implicated in many cancers, not only does it count as members many oncogenes, at the top of the pathway lies the most potent breast cancer oncogene known to date – receptor tyrosine kinase HER2 (also known as ERBB2) – a key receptor at the top of the signal transduction chain.

**Figure 3:**
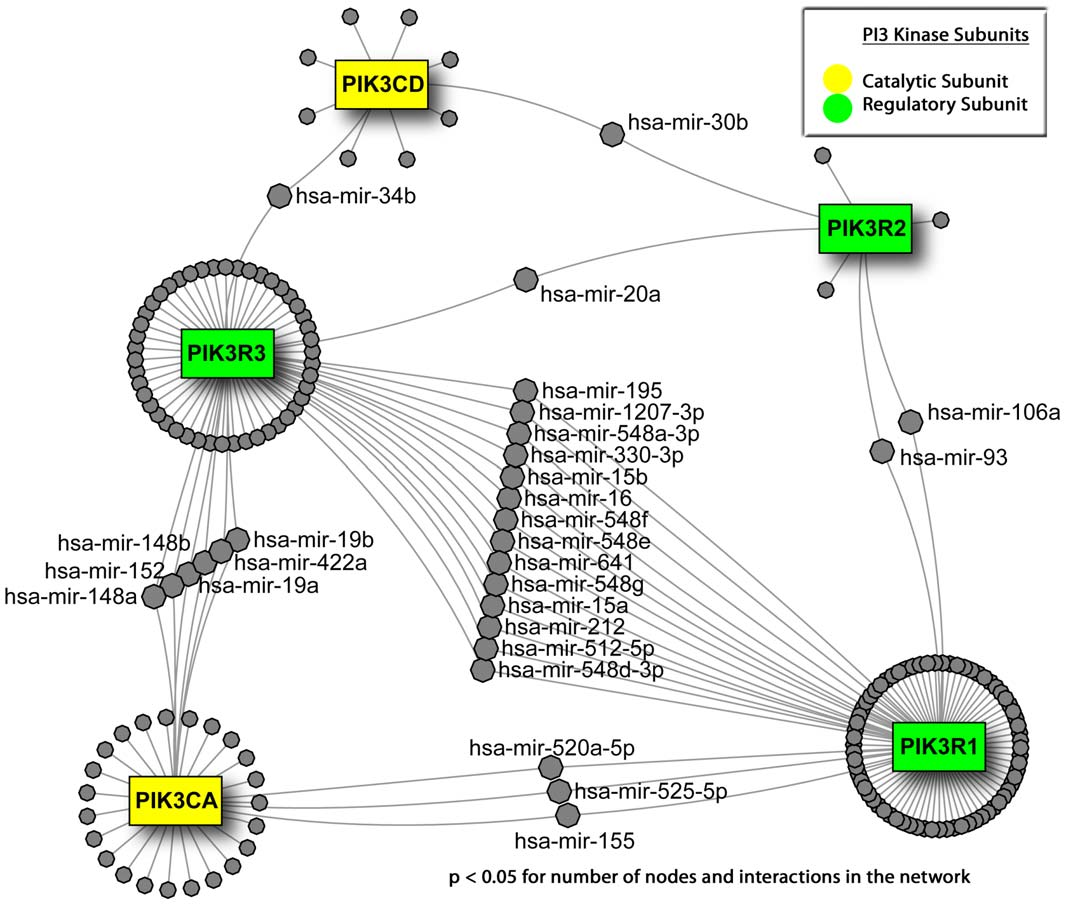
MicroRNA interaction network for assembly of PI3K subunits. Mapping PI3K subunits to microRNA interactions produces a network that is significantly more connected than at random (p<0.05). Green nodes are regulatory subunits and yellow nodes are catalytic subunits.

The PI3K family is divided into 3 classes. Members of each class of PI3K molecules comprise 2 subunits – a regulatory subunit and a catalytic subunit. These subunits are distinct proteins coded in different regions of the genome as either distinct genes or splice variants transcribed out of a similar locus producing translated proteins of varying sizes. The particular assembled combination of the 2 subunits of PI3K determine the molecule's structure and function, and varying combinations of subunits are active in entirely different cellular settings [69]. Using interactions at the 3+DB robustness level, we map the microRNAs targeting genes involved in the assembly of Class 1 PI3K (Figure 3). Immediately, it becomes evident that the possibility for PI3K subunit regulation at a post-transcriptional level is real. The network resulting from the input of all Class 1 PI3K subunit genes (PIK3CA/B/C/D, PIK3R1/2/3/4/5/6) contains five primary nodes (the other subunit genes are missing due to the lack of microRNAs targeting them in a sufficient number of databases), 181 secondary nodes and 206 interactions. Permutation analysis of randomly selected 5-node networks confirmed that this provides a significant enrichment (p<0.05) for number of nodes and interactions in the network. The most striking feature of the network is the participation of primary nodes in interactions with at minimum two other nodes – indicating that this network is significantly more connected through microRNAs than one would expect by chance alone (p<0.01).

#### Regulation of PI3K signalling

**Figure 4:**
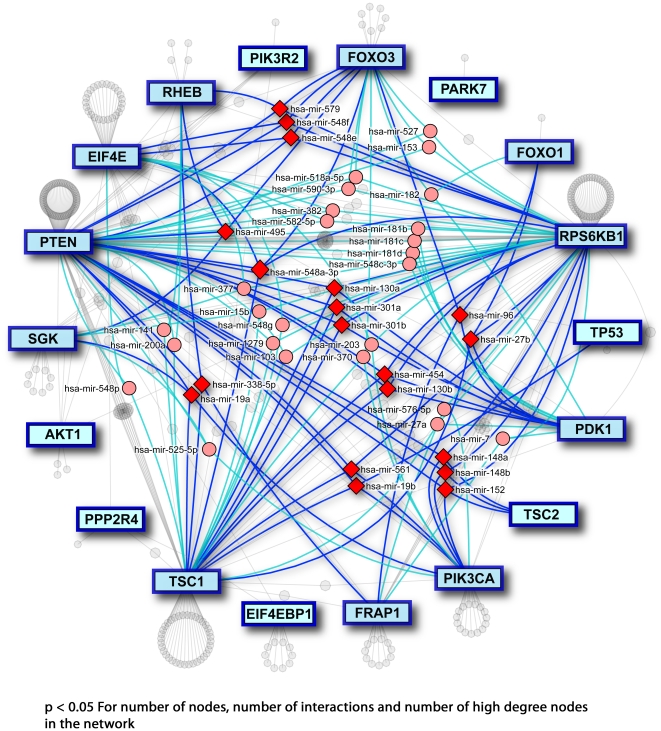
MicroRNA interaction network for elements of the PI3K pathway. Mapping the elements of the PI3K pathway based on a literature review [66], produces a network where many genes are targeted by common microRNAs suggesting a novel microRNA role of pathway regulation.

To further examine microRNA involvement in this pathway, we use a model of the downstream signalling components of this pathway as indicated in a recent review [66]. Here we unveil a second highly-connected microRNA network (Figure 4) (based on 2000 permutations: p<0.05 for number of nodes in the network, p<0.05 for number of interactions in the network, p<0.05 for number of nodes with degree ≥4). It is quite surprising to see the number of microRNAs that can co-target potent tumour suppressors and oncogenes. We find a microRNA – hsa-mir-19b – that concurrently targets PTEN-TSC1-PI3KCA-TP53, and others that co-target RPS6KB1-PDK1-TSC1-PTEN and PTEN-RPS6KB1-FOXO3-TSC1. In addition, there are many microRNAs that target pairs of elements of this pathway: 15 microRNAs target RPS6KB1 and PTEN, 8 microRNAs target both RPS6KB1 and TSC1, and 4 microRNAs target both EIF4E and RPS6KB1. Clearly, we are only beginning to understand the level of regulation possible with microRNAs co-targeting many different genes, but it is becoming increasingly evident that this level of network complexity governs some interesting and previously hidden relationships between potent oncogenes and tumour suppressors in the cell.

### Examination of KEGG and Reactome pathway-based microRNA networks

#### Basic Network Parameters

**Figure 5:**
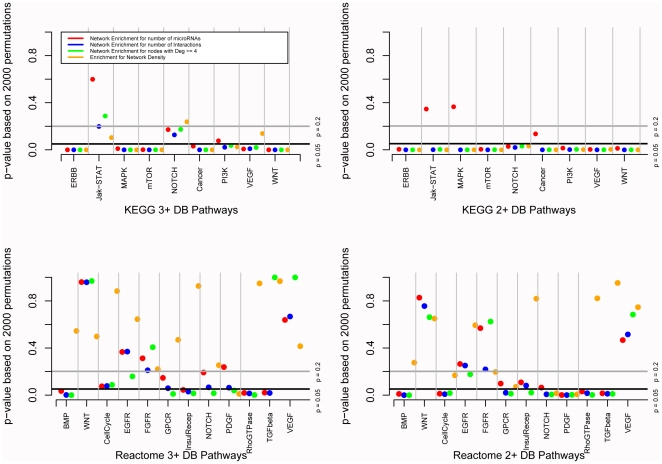
MicroRNA interaction network characteristics. Examination of four microRNA interaction network characteristics across well-known signalling pathways using KEGG (panels A and B) and Reactome pathway databases (panels C and D). Signalling pathways tend to be enriched for the number of microRNAs, the number interactions and the number of high degree nodes mapped.

After initially testing our hypothesis on the PI3 Kinase pathway, we decided to undertake a more extensive and rigorous examination of signalling pathways within the cell. Since pathway definitions have not been unanimously settled and there is still much debate as to which resource defines a signalling pathway most accurately and comprehensively, we decided to use pathways delineated by the Kyoto Encyclopedia of Genes and Genomes database (KEGG) [70], [71] and pathways defined by the Reactome [72], [73], [74] database to further support the microRNA networks built based on expert-curated pathway reviews in the previous section. Examining interactions predicted at 2 threshold levels: 2+DB and 3+DB, we created microRNA networks for both the canonical signalling pathways and for 2000 permutations of pathways created with the same number of primary node genes. Our findings showed a similar trend for most interaction sets and signalling pathways that we examined. We found that true signalling pathways tend to involve more microRNAs and contain more interactions, as well as having more high degree nodes (degree ≥4) than pathways created out of a random set of starting nodes. We examined 9 KEGG pathways and 12 Reactome pathways at the 2+DB and 3+DB interaction thresholds. The pathways with the lowest average p-values (that is the average of p-values across the 4 measured parameters - number of network interactions, number of network microRNAs, number of network nodes with degree ≥4 and network density) were KEGG pathways: ERBB signalling pathway (hsa04012) (2+DB), mTOR signalling pathway (hsa04150) (2+DB), Wnt signalling pathway (hsa04310) (2+DB), MAPK signalling pathway (hsa04010) (3+DB) and Pathways in cancer (hsa05200) (3+DB) with average p-values of p<0.0006, p<0.0009, p<0.002, p<0.002, p<0.007, respectively (Figure 5). Of the pathways described in both the KEGG and Reactome databases (NOTCH, VEGF and WNT), WNT results were the least conserved across both databases – showing significance in KEGG (average p-values of p<0.002 and p<0.036 for 2+DB and 3+DB respectively), but not in Reactome (average p-values of p<0.64 and p<0.68 for 2+DB and 3+DB respectively), while NOTCH measured parameters were the most likely to be consistent across the two databases (average p-values of p<0.102 and p<0.105 for 2+DB and 3+DB respectively in KEGG and average p-values of p<0.256 and p<0.139 for 2+DB and 3+DB respectively in Reactome). We found that some pathways had greater tendencies than others to show significance – for example the FGFR and Cell Cycle Genes pathways (which, it could be argued, is not a signalling pathway and hence does not fit within this study and hence acts as our negative control) described only by the Reactome database had a tendency towards higher p-values than other pathways examined (Reactome FGFR pathway average p-values of p<0.35 and p<0.4 for 2+DB and 3+DB respectively and Reactome Cell Cycle Genes average p-values of p<0.78 and p<0.45 for 2+DB and 3+DB respectively). The measured parameters found to be most frequently significant across all studied scenarios were the number of microRNA nodes in the network with degree ≥4 (significant at p<0.05 in 30/42 tested scenarios), and the number of total microRNA:target interactions in the network (significant at p<0.05 in 27/42 tested scenarios). As highlighted in Figure 5 – one can find enrichments that are supported by both pathway databases, while other enrichments are highlighted in the analysis using one or the other pathway database. Examining expert-curated pathways, KEGG pathways and Reactome pathways with similar findings gives us confidence that this phenomenon is in fact real.

#### Centrality Measures

We further examined network betweenness centrality (using Brandes' algorithm [75] in R [76] using the RBGL package [77], [78]) as well as the average betweenness centrality of the top 10 genes and microRNAs by degree, and the average shortest path length between the top 10 genes. In general, these measures were not found to be significantly different in true signalling pathways from the random networks across most pathways. For the KEGG 3+DB signalling pathways network betweenness centrality – a measure of the difference between the node with the highest betweenness centrality (the node on the most shortest paths) to all other nodes in the network – we did see a small trend towards pathway networks having lower betweenness centrality (p<0.0001 (WNT pathway) to p<0.837 (VEGF pathway). This trend suggests that true signalling pathways have a more balanced centrality structure with fewer “hub” nodes than random networks do. However, we did not see any difference in the betweenness centrality of the top ten microRNAs by degree or the top ten genes by degree in the signalling pathways (p<0.089 to p<0.687 for microRNAs and p<0.37 to p<0.987 for genes). Further, due to the distributions of the network values for average and maximum shortest paths (measured with Dijkstra's algorithm [79]) between the top 10 genes we were unable to conclusively evaluate these parameters (95% of average shortest path values were 3 and almost 75% of maximum shortest path values were infinite). This lack of conclusive significance in centrality measures can be explained by the fact that we did not model interactions between proteins in our networks, choosing to examine only interactions between genes and microRNAs. Thus, our networks tended to have a particular structure requiring all pathways to alternate between gene and microRNA due to the lack of protein-protein connections. Integration of protein-protein interactions with microRNA-target interactions in a network could be re-examined at a later date.

#### Network Hubs

We also examined the possibility that hubs in these microRNA networks might be more likely to be date or party hubs as defined in Han et al.'s paper [80]. Using our I2D database [81], [82] we examined known human protein-protein interactions for a binomial distribution to define such hubs, and failed to find such a distribution, hence we are unable to further study any such relationship.

#### Universe and Intra-pathway microRNAs

**Figure 6:**
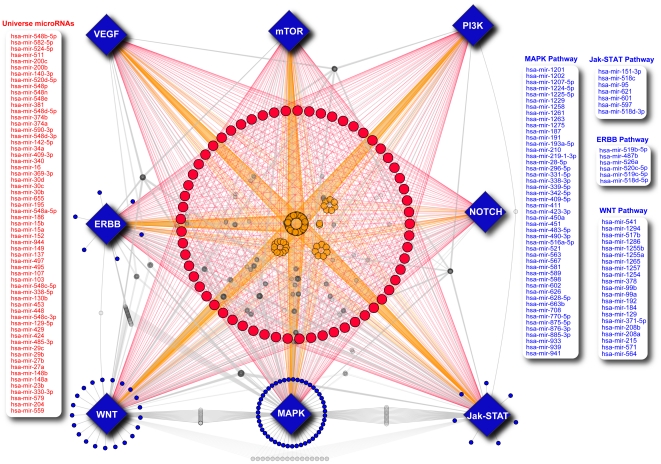
Micronome Roadmap. Network based on KEGG signalling pathways built on 3+DB microRNA interaction data. Universe microRNAs are shown in red and intra-pathway microRNAs are in blue.

**Figure 7:**
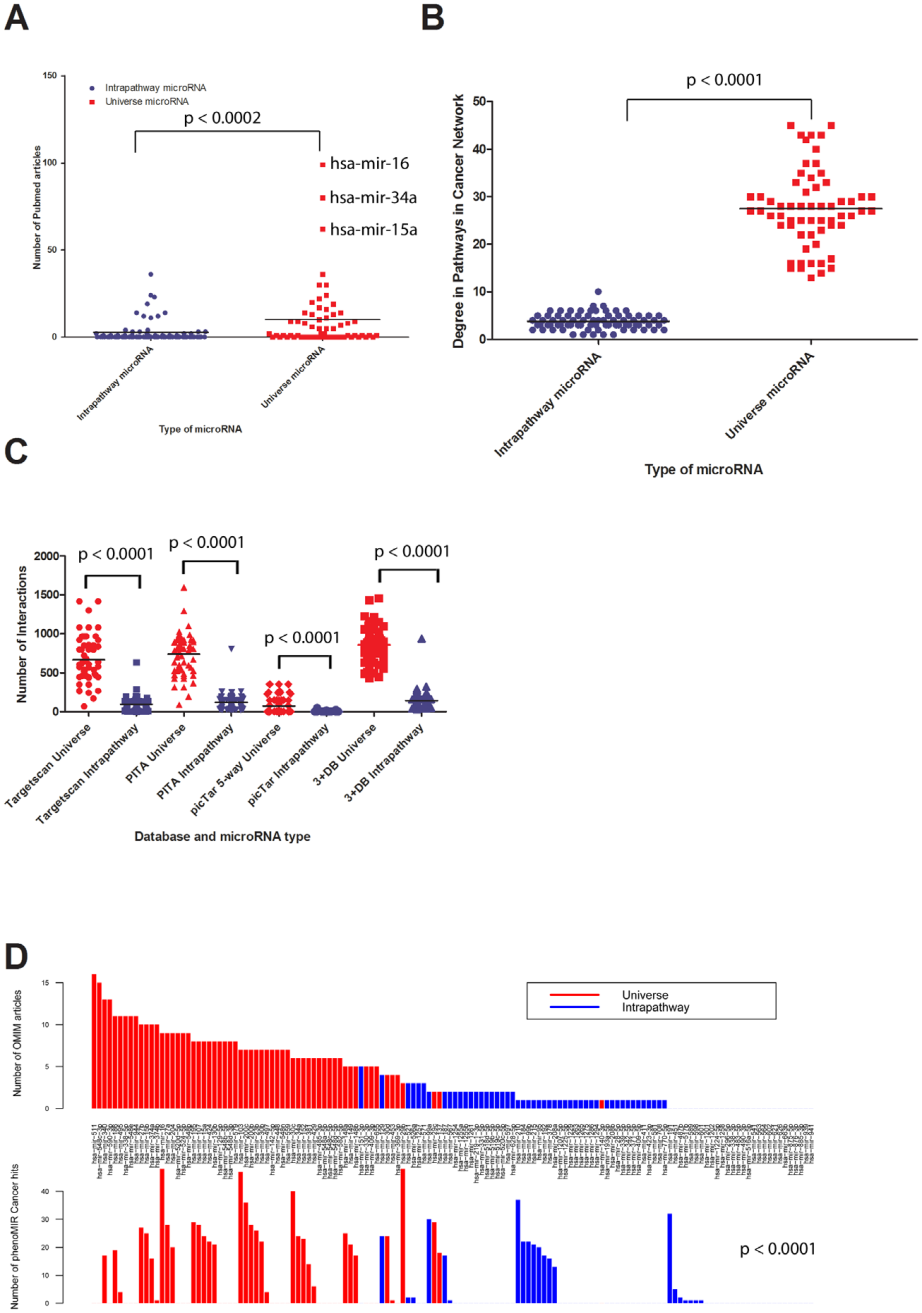
Comparison of universe and intra-pathway microRNAs. Panel A: Universe microRNAs have a significantly larger number of PubMed papers compared to intra-pathway microRNAs (p = 0.0002). Panel B: Universe microRNAs have significantly higher degree in the KEGG Pathways in Cancer 3+DB network (p<0.0001). Panel C: Universe microRNAs have significantly more predicted target interactions than intra-pathway microRNA across several different microRNA prediction databases (p<0.0001). Panel D: Top – Universe microRNA targets (red) tend to have more OMIM hits than intra-pathway microRNAs (blue). Bottom – Universe microRNAs themselves have more “cancer” PhenomiR hits than intra-pathway microRNAs (p<0.0001), supporting the result in panel B.

Upon realizing that microRNAs play a large role within signalling pathways – we produced a road map to delineate the inter-pathway connections (Figure 6). It quickly became clear that there are distinct classes of microRNAs. Examining microRNAs with degree greater than two in any signalling pathway, we were able to identify 77 microRNAs that act only in an intra-pathway manner, affecting multiple targets but only within one single pathway. These microRNAs tend to target the ERBB, mTOR, MAPK, WNT and Jak-STAT pathways and no intra-pathway microRNAs appear to target the VEGF, NOTCH and PI3K pathways. We further identified 61 microRNAs targeting all 8 KEGG pathways that we examined at the 3+DB level. In attempts to validate this classification of microRNAs into intra-pathway and universe classes, we went to the literature. Searching for total PubMed articles, we see a significant difference between universe and intra-pathway microRNAs (p<0.0002) – with universe microRNAs discussed more frequently (Figure 7A). Further, the most discussed microRNAs, hsa-mir-15a, hsa-mir-16 and hsa-mir-34a have high degree in the many pathways in which they are involved (hsa-mir-15a has intra-pathway ranking of 2(ERBB), 1(Jak-STAT), 2(MAPK), 3(VEGF), 4(mTOR), 1(WNT), 27(NOTCH)). This observation makes sense when one considers that many decisions regarding the selection of microRNAs to study are based on high-throughput experiments, through over-expression of a library of microRNAs and examination of several simple read-out conditions. It follows that microRNAs with involvement in many pathways – universe microRNAs – might be able to produce large changes within the cell, resulting in measurable outcomes compared to controls. As such, these microRNAs might be selected for further study, resulting in more PubMed articles. When constructing the microRNA road map from known signalling pathways in KEGG, we did not include the Pathways in Cancer gene network, since it is not a signalling pathway in its own right. Overlaying universe and intra-pathway microRNAs with the Pathways in Cancer Network built for Figure 5, we see that universe microRNAs have much higher degree than intra-pathway microRNAs in the Pathways in Cancer network (p<0.0001) (Figure 7B). Considering that this type of effect could have been induced by our filtering methods, we examined our 3+DB interaction set for the number of targets predicted for both universe and intra-pathway microRNAs. We did see significantly more predicted targets for universe microRNAs than for intra-pathway microRNAs (p<0.0001). However, this distribution was replicated in TargetScan predicted targets (p<0.0001), PITA predicted targets (p<0.0001) and picTar predicted targets (p<0.0001) (Figure 7C). Since this distribution transcends any filters that we have applied and it holds for these individual database prediction sets we suggest that universe microRNAs simply tend to have more targets, and are therefore able to exert a broader program of control over the cell than are intra-pathway microRNAs.

At this point, we would like to address the issue of bias in the data and distinguish microRNA interaction sets from protein interaction datasets. There is one large and obvious difference between protein and microRNA interactions. Protein-protein interactions are often curated through highly-biased information gathering methods; literature searches, which are biased towards highly-studied proteins, and high-throughput experiments focusing on finding all partners for one protein of interest, while considering a library of potential partners. Although useful interaction generating techniques, they cannot be relied open to uncover protein-protein interactions evenly across the proteome. MicroRNA:target relationships are different. The information upon which our study is built is entirely sequence-based. The databases considered do use different algorithms to make their predictions; however, the predictions are free from bias due to the ground truth that everything studied is sequence-based. Conservation of a binding site, binding site accessibility and presence or absence of a seed-region depend entirely on the coded gene, its transcribed RNA and the sequence of the microRNA that might bind to it, freeing us from the requirement to compensate for any bias in microRNA:target predictions. That being said, one possible bias that we cannot decouple from our current analysis is the relationship of the length of a given gene's 3′ UTR and the number of microRNAs that target it. It remains unclear if the fact that genes with long 3′ UTRs tend to have more predicted targeting microRNAs is due to the fact that this is the way that the biology works or if it is simply related to the odds of having more binding sites in longer UTRs.

Finally, we examined the differences between universe and intra-pathway microRNAs in a disease setting. First, we examined the cumulative number of Online Mendelian Inheritance in Man (OMIM) database [83] hits for all targets of each microRNA (Figure 7D Top Panel). Arranging them in decreasing order we show that universe microRNAs have many more OMIM hits than do intra-pathway microRNAs. It should be noted here that we did not normalize for number of targets per microRNA. The lower panel shows the universe microRNAs having significantly more cancer hits for each microRNA in the PhenomiR database [84] compared to intra-pathway microRNAs (p<0.0001). We see a strong distinction between universe microRNAs and intra-pathway microRNAs for disease association, again supporting our hypothesis that universe microRNAs are a subset of microRNAs that can target many genes within the cell, acting as master controls.

As further explanation for why universe microRNAs have been more studied than intra-pathway microRNAs, a comparison of microRNA “number” - their unique identifying IDs, which were assigned in approximate order of discovery - shows that universe microRNAs have a lower average identifying number than intra-pathway microRNAs (mean ID for universe microRNAs = 51 vs intra-pathway microRNAs = 84, p<0.0001).

**Figure 8:**
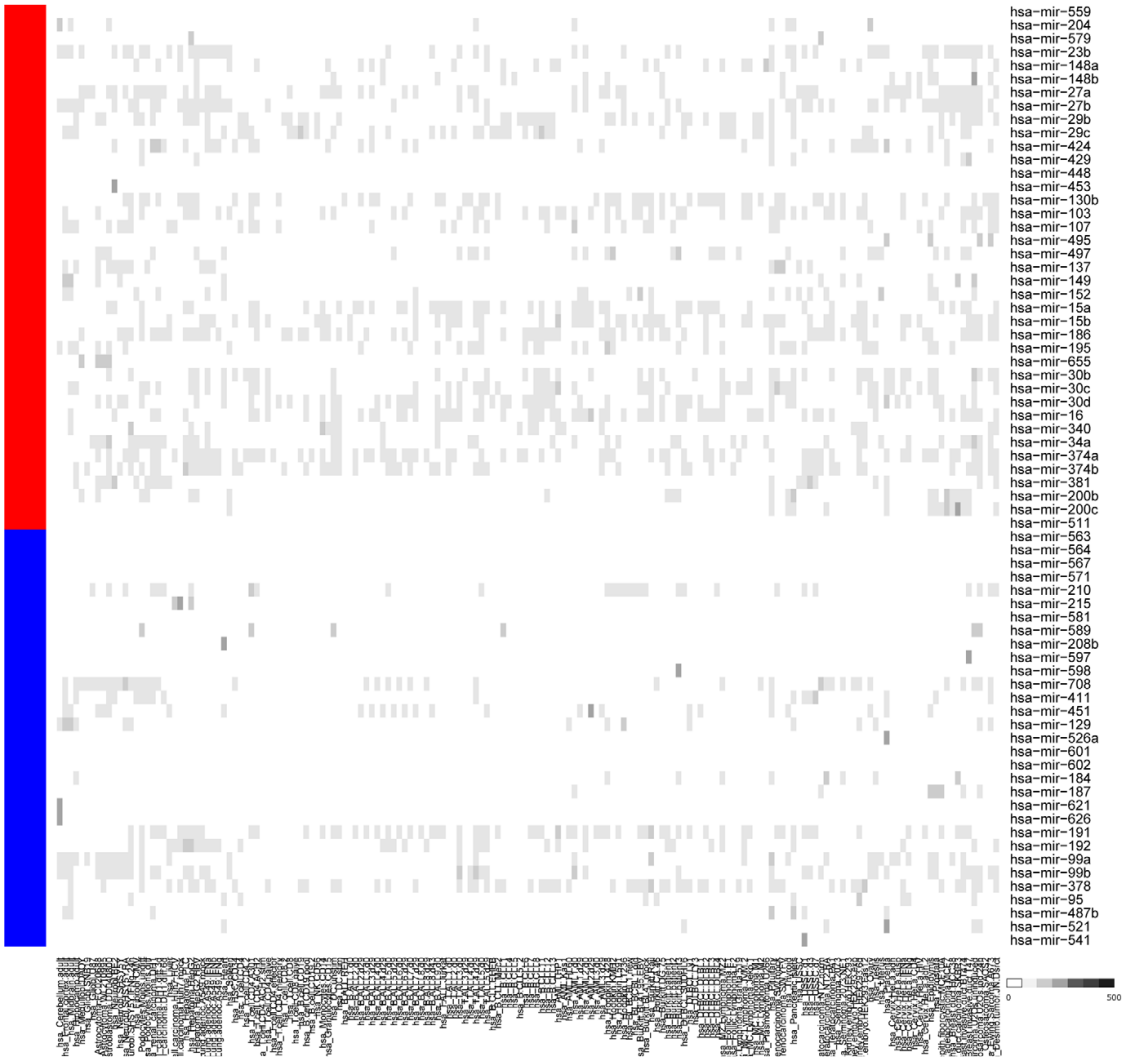
Expression of universe and intra-pathway microRNAs. Universe microRNAs are expressed in a broader panel of tissues than intra-pathway microRNAs [96].

This may be either because universe microRNAs have been discovered earlier purely by chance and thus were more studied, or they may truly be more universal and thus were easier to discover under many conditions. To provide additional evidence to answer this question, we considered expression of microRNAs across a panel of tissues from Landgraf et al. [85]. Figure 8 shows a heatmap comparing universe and intra-pathway microRNA expression across tissues, confirming that universe microRNAs are more widely expressed than intra-pathway microRNAs. Thus, it is more likely that universe microRNAs are more broadly affecting varying cell types, and through their misexpression, universe microRNAs have the opportunity to create a more global change quickly by affecting genes in many different pathways. To further understand their role in human disease thus warrants further research.

This work in microRNA interaction networks provides more evidence for the possibility that microRNAs are in fact working in a coordinated fashion with each other and within signalling pathways. It has been previously noted that many microRNAs might co-bind to a UTR [2], [31], [48], [85], and perhaps our results support that view, since genes in a common pathway share many more common microRNAs than one would expect by chance (p<0.0035 to p<0.365 for KEGG 2+DB). This thinking opens the door for many exciting *in vitro* experiments to examine this co-regulation and co-binding, and raises the questions, how many microRNAs might actually be occupying a 3′ UTR at once? Is it a sequential or a parallel microRNA process? Future work to determine the layout of such microRNA binding sites in the untranslated regions might provide further insight here – and a within pathway study of the degree of overlap and layout of microRNA binding sites on interacting genes would provide insight into the microRNA regulatory network. Another interesting expansion of this work would be to determine predicted binding sites in 5′ UTRs and coding regions of target genes, and integrate them with RNA22 predictions already included in mirDIP to allow cross-database comparisons. While the majority of confirmed microRNA binding sites fall into 3′UTRs, fuctional binding sites have been shown in other regions [86], [87], [88] and attempts to include them in mirDIP would result in a more complete representation of true microRNA target genes within the cell.

The identification of two distinct classes of microRNAs – universe and intra-pathway microRNAs – lays the frame work for possibly hierarchical organization of pathway- and gene-level control and execution of gene regulation. Using PhenomiR, we provide the first disease-associated evidence that universe microRNAs may be more likely to be involved in cancer specifically – showing significantly more involvement in breast (p<0.07), ovarian (p<0.005) and lung cancers (p<0.05) and in carcinogensis overall (p<0.0001) while also showing involvement in human disease in general (p<0.0001), and this information will allow us to focus our disease-driven microRNA-associated research towards a smaller subset of these potent cellular regulators.

### Conclusions

#### MicroRNA Prediction Databases

Similar to work done by other groups, we have examined microRNA prediction databases to determine that PITA Top, picTar 5-way and Targetscan Conserved provide the most accurate microRNA:target predictions. Using different prediction algorithms, individual predictions overlap only partially and they differ in their precision and recall when compared to *in vitro* truth data. However, each has a particular application where it might be best suited for use. We have further examined the importance of filtering target predictions before making microRNA database comparisons, and have determined that filtering by both experiment cell type and microarray chip type are crucial steps that alter gene prediction sets by up to 40%. We suggest that when searching for true microRNA targets, it is useful to consider such steps.

#### mirDIP

We have presented a unique database to aid researchers in determining the optimal microRNA prediction databases to use for application-specific microRNA:target searches. mirDIP allows users to focus their searches on any subset of microRNA prediction databases, in either “high precision” or “high recall” databases depending on their path of study.

#### Discovery of Universe and Intra-pathway microRNAs in Interaction Networks

Using data from mirDIP, we found that microRNAs are significantly more involved in known signalling pathways compared to random chance, producing networks with more interactions (p<0.1 in 76% of tested pathways). Signalling pathways contain many microRNAs that target multiple elements of the pathway, perhaps suggesting a level of transcriptional regulation not previously described. Our data suggest a possible co-regulation of signalling proteins at the post-transcriptional level – whether concurrent or sequential – which opens new line of research to study hierarchical organization of microRNAs. Further, we have identified two novel classes of microRNAs: universe and intra-pathway microRNAs, which are significantly differentiated by the degree of their involvement in signalling pathways within the cell and their association with cancer (p<0.0001) and human disease (p<0.0001). Universe microRNAs are involved in regulation of many known signalling pathways, while intra-pathway microRNAs are pathway-specific and do not appear to play a global role in cellular regulation.

## Materials and Methods

MicroRNA predictions were downloaded from the individual microRNA prediction sites:

- http://microrna.org (Sept. 2008)
- http://microrna.sanger.ac.uk/targets/v5/(ver. 5) (now http://ebi.ac.uk/enright-srv/microcosm/htdocs/targets/v5/)
- http://genie.weizmann.ac.il (ver. 6 Aug. 2008)
- http://cbcsrv.watson.ibm.com/rna22.html (Aug. 2007)
- http://targetscan.org (Release 5.0 Dec. 2008)
- http://pictar.mdc-berlin.de/(Mar. 2007)
- http://diana.cslab.ece.ntua.gr/microT/(V3.0)

### Target Prediction Files

All target prediction files were processed to contain the same information in the same format. The UCSC Genome Browser (http://genome.ucsc.edu/) [89], [90], [91] and Galaxy [92], [93] were used to convert all files to include HUGO gene names for all interactions according to human genome version hg18. RNA22, picTar and DIANA microT required intermediate mapping steps using Ens54 [94] and RefSeq May 2006 [95] assemblies. All files were then combined to produce one file of all predictions. A filtering step produced the interaction files for NAViGaTOR – eliminating all interactions present in less than 2 or 3 microRNA prediction databases. To avoid double-counting interactions present in 2 databases from the same source compiled with different stringency requirements, only the most stringent PITA and picTar microRNA prediction files were used as inputs into the integration and filtering steps.

### microRNA microarray Truth Files

Files used to compare microRNA prediction files to truths were obtained from the following GEO Datasets: GSE2075 [57], GSM306946 [53], GSE6838/GSM155064 [58], GSE6207 [60], GSM302945 [53], GSE13296 [54], GSE6838 [58], GSM302995 [53], GSE12091 [55], GSE9586 [59], GSE9742 [56], GSE14507, GSE12092 [55]. Thresholds for low-, med- and high- confidence truths were established using p-values of p<0.1, p<0.05, p<0.01, where replicates were present, and otherwise at three step-wise incremental knockdown or over-expression thresholds dependent on the distribution of target knockdown – 50%-25%-10% for mir-335 (GSE9586) and mir-7 (GSE14507) (in this case – since there were only 2 replicates, we also required the replicates to be within 15% of each other), 75%-25%-10% for mir-155 (GSE13296) and 25%-20%-10% for mir-124 (GSE6207).

### Target Filtering

To filter target predictions prior to prediction database comparison, we used genes present in the bottom quartile of the control cell line microarray experiment. In most cases, one or more negative control sample values were present and those values were averaged and then ranked by intensity value. When filtering by experimental cell type, only genes not present in this bottom quartile passed through our filter. In the few cases where it was not possible to extract the control cell line values from the experiment (mir-1 Lim et al. [5] and mir-124 Lim et al. [5]), filtering genes from the negative controls from a microarray experiment in the same cell line were used (mir-98 negative controls Gennarino et al. [53]). We further filtered by the presence of the predicted target gene on the microarray chip used in the experiment, information available at GEO datasets.

### NAViGaTOR Networks

NAViGaTOR networks [81], [82] were built based on the microRNA:target interaction files discussed above, with two levels of robustness: interactions present in two or more databases (2+DB) or interactions present in three or more databases (3+DB). Note that out of the eleven databases examined in the first section, only nine are used for the microRNA interaction networks due to the fact that PITA Top Targets (used) is a subset of PITA All Targets (not used) and picTar 5-way (used) is a subset of picTar 4-way (not used). Using groups of Associated Genes of interest (as determined by well known sub-units [69], pathways extracted from the literature [66] and KEGG [70], [71] and Reactome [72], [73], [74] databases) as primary nodes – networks were built to examine the interactions between the given associated gene set at the microRNA level. Associated gene network significance was evaluated based on four characteristics: 1) the number of nodes in the network, 2) the number of interactions in the network, 3) the number of nodes with degree greater than three, and 4) the measured network density, and compared to values obtained from 2000 random networks constructed from the same number of primary nodes (genes randomly selected from the interaction file, hence genes that have been identified as participating in a microRNA interaction by at least two or three prediction databases). KEGG pathway HUGO IDs were used to create networks, while Reactome Swiss Protein IDs were mapped in the UCSC Genome Browser to HUGO IDs before networks were built. Networks were built using the graph (ver. 1.24.1) [78] and RBGL (ver. 1.22.0) packages [77] of the R Statistical Package software (ver. 2.8.1) [76]. When comparing pathways represented in both KEGG and Reactome databases, comparisons were made between the differences of the sums of the p-values of the four network parameters. All analysis was done using NAViGaTOR ver. 2.1.13 [1] (http://ophid.utoronto.ca/navigator).

### Examination of Date and Party Hub Nodes

In our examination of human protein-protein interactions to determine whether a bimodal date and party hub distribution was present, I2D human source interactions were used [81], [82].

### Universe and Intra-pathway microRNAs

Using NAViGaTOR ver. 2.1.13 to display the microRNA:pathway interactions from the KEGG 3+DB study, we laid out the micronome roadmap to identify universe and intra-pathway microRNAs. Comparisons between the two classes of microRNAs and number of associated PubMed articles were done using biopython (v1.50) (http://biopython.org). OMIM [83] hits and PhenomiR (v1.0) [84] hits were drawn from their respective sources (http://www.ncbi.nlm.nih.gov/omim/(accessed Feb. 2010) and http://mips.helmholtz-muenchen.de/phenomir/).

### Tarbase Comparison

We used Tarbase V5.0 [63] to compare our 2+DB interaction set to the best curated set of microRNA interactions existing. We used only human interactions, eliminated the support_type = FALSE interactions and mapped by the HGNC column.

Details about mirDIP can be found in Methods S1.

## Supporting Information

File S1 Descriptions of the mirDIP interface and sample mirDIP searches.
(DOC)

Figure S1 Key component fields of the microRNA data integration portal.
(TIF)

Figure S2 Sample mirDIP search for microRNAs targeting one gene of interest, requesting high precision target data.
(TIF)

Figure S3 Sample mirDIP search for microRNAs co-targeting three genes of interest using data from one individual microRNA database.
(TIF)

Figure S4 Sample mirDIP search for targets of one particular microRNA, selecting a microRNA prediction algorithm based on specific algorithm criteria.
(TIF)

Figure S5 Sample mirDIP search for microRNAs targeting one gene of interest using targets predicted by 4 or more microRNA prediction databases.
(TIF)

Methods S1 mirDIP development.
(DOC)
