# Supplementary material for: NAViGaTing the Micronome – Using Multiple MicroRNA Prediction Databases to Identify Signalling Pathway-Associated MicroRNAs
Source: PLoS One. 2011 Feb 25;6(2):e17429. doi: 10.1371/journal.pone.0017429 (PMC3045450; doi:10.1371/journal.pone.0017429)
Supplement: File S1 — Descriptions of the mirDIP interface and sample mirDIP searches. (DOC) [file pone.0017429.s001.doc]

mirDIP – Interface Description

Supplemental Figure 1 shows the key component fields of the mirDIP interface.

1. **Gene Symbol Search Box** – used to search for a gene.
2. **MicroRNA Search Box** – used to search for a microRNA.
3. **Normalized Score Range Search Boxes** – used to search for genes or microRNAs with a particular score range.
4. **Source Filter Box** – used to specify which microRNA prediction database(s) should be used in a search.
5. **Prediction Features Box** – used to select specific microRNA prediction database(s) based on characteristics of the prediction algorithms behind them.
6. **Quality Filter Box** – used for supporting multiple scenarios during searching: a) identifying only highly likely predictions, b) identifying all possible predictions, c) optimized balance of both strategies. See the mirDIP Instructions tab for a more detailed discussion.
7. **Database Occurrences Box** – used to specify how many databases must support all returned predictions.
8. **Intersecting IDs Box** – used when searching for multiple genes or microRNAs at once, to ensure that only an intersection of predictions is returned. Can be used with the Source Filter or Quality Filter Boxes.
9. **Search Button** – used to begin a search.

Using mirDIP – Four examples

**1/ The user is interested in the gene PTEN. They would like to do some in vitro experiments and would like to identify high precision (high certainty) microRNAs that target PTEN.**

## Steps:

Enter PTEN in the Gene Symbol box

Choose the High Precision check box under Quality Filter

Click Search

To sort results by normalized score, click on the black score(norm) heading. Click again to sort in the reverse direction

See Supplemental Figure 2 for the Search Setup

**2/ The user is interested in the genes PTEN, TP53 and TSC1. They would like to find microRNAs predicted by microRNA.org that might co-target all three genes.**

## Steps:

Enter PTEN TP53 and TSC1 in the Gene Symbol Box

Click microRNA.org in the Source Filter Box

Click the Intersecting IDs box

Click Search

To sort results by normalized score, click on the black score(norm) heading. Click again to sort in the reverse direction

See Supplemental Figure 3 for the Search Setup

**3/ The user is interested in the microRNA hsa-mir-22. They would like to examine some predicted targets from sources that consider conservation as well as site accessibility.**

## Steps:

Enter hsa-mir-22 in the microRNA

Click Conservation and Site Accessibility in the Prediction Features Box

Click Search

To sort results by normalized score, click on the black score(norm) heading. Click again to sort in the reverse direction

See Supplemental Figure 4 for the Search Setup

**4/ The user is interested in the gene TP53. They would like to examine microRNAs predicted to target TP53 that occur in at least 4 microRNA prediction databases.**

## Steps:

Enter TP53 in the Gene Symbol

In the Database Occurrences box, select 4 from the drop down list

Click Search

To sort results by normalized score, click on the black score(norm) heading. Click again to sort in the reverse direction

See Supplemental Figure 5 for the Search Setup
