# Supplementary figures and images for: NAViGaTing the Micronome – Using Multiple MicroRNA Prediction Databases to Identify Signalling Pathway-Associated MicroRNAs
Source: PLoS One. 2011 Feb 25;6(2):e17429. doi: 10.1371/journal.pone.0017429 (PMC3045450; doi:10.1371/journal.pone.0017429)

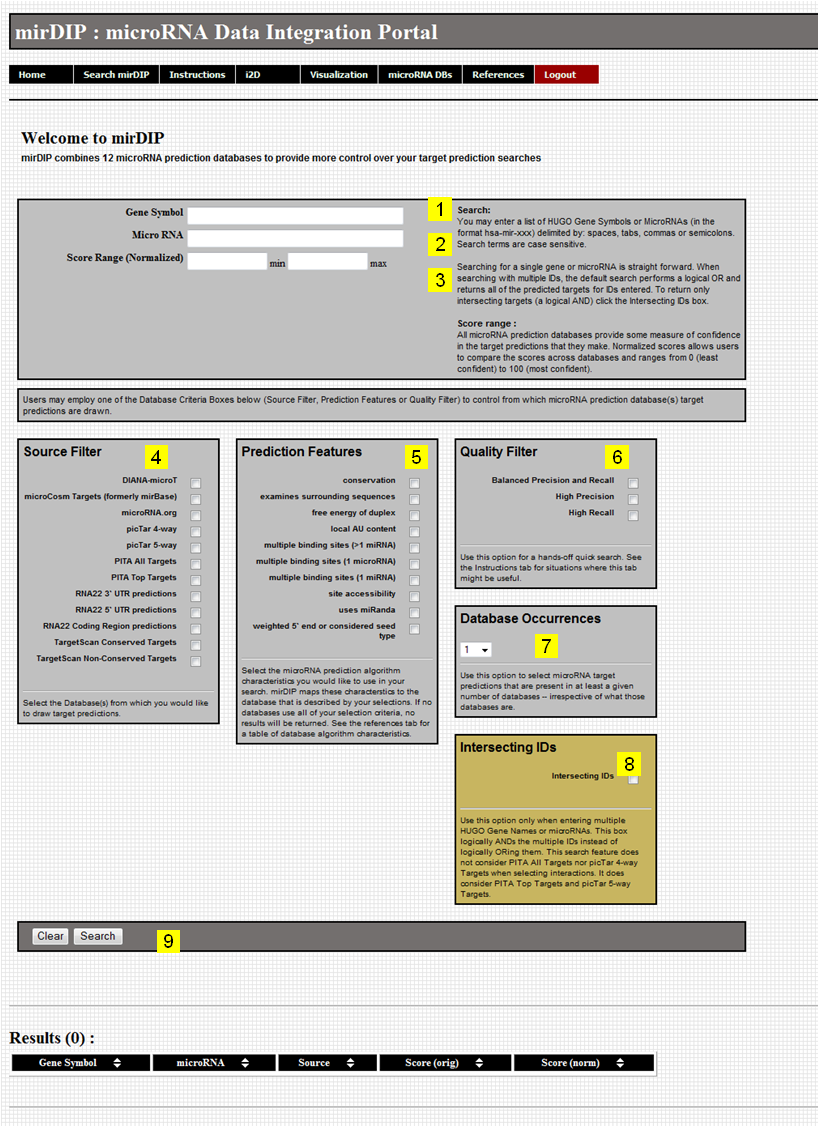

Supplement: Figure S1 — Key component fields of the microRNA data integration portal. (TIF) [file pone.0017429.s002.tif]

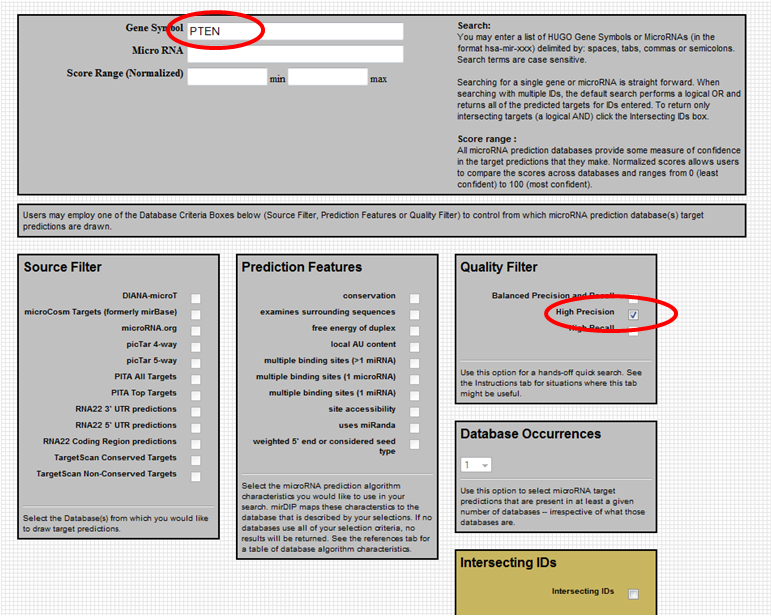

Supplement: Figure S2 — Sample mirDIP search for microRNAs targeting one gene of interest, requesting high precision target data. (TIF) [file pone.0017429.s003.tif]

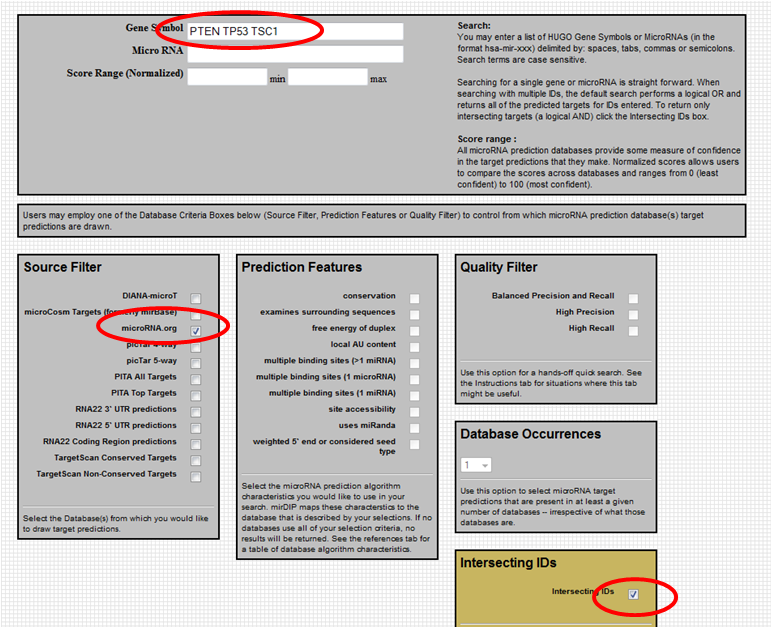

Supplement: Figure S3 — Sample mirDIP search for microRNAs co-targeting three genes of interest using data from one individual microRNA database. (TIF) [file pone.0017429.s004.tif]

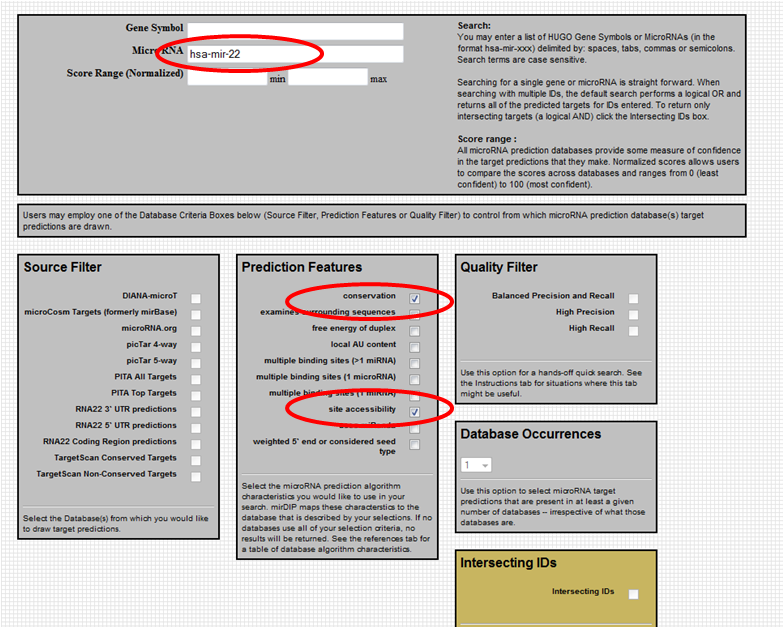

Supplement: Figure S4 — Sample mirDIP search for targets of one particular microRNA, selecting a microRNA prediction algorithm based on specific algorithm criteria. (TIF) [file pone.0017429.s005.tif]

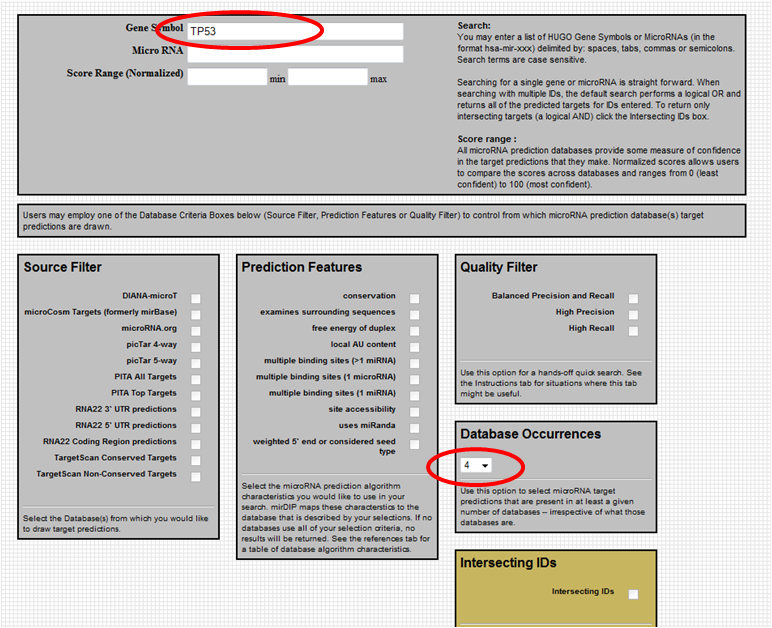

Supplement: Figure S5 — Sample mirDIP search for microRNAs targeting one gene of interest using targets predicted by 4 or more microRNA prediction databases. (TIF) [file pone.0017429.s006.tif]
