# Supplementary material for: NAViGaTing the Micronome – Using Multiple MicroRNA Prediction Databases to Identify Signalling Pathway-Associated MicroRNAs
Source: PLoS One. 2011 Feb 25;6(2):e17429. doi: 10.1371/journal.pone.0017429 (PMC3045450; doi:10.1371/journal.pone.0017429)
Supplement: Methods S1 — mirDIP development. (DOC) [file pone.0017429.s007.doc]

***Supporting Methods***

The mirDIP portal was developed in Java. We used IBM DB2 ver. 9.5 database back-end and deployed it using IBM WebSphere 6.1 web server (<http://ophid.utoronto.ca/mirDIP> ). Our application server has 32 IBM Power5 processors at 2.3 GHz each with 263 GB of RAM. Our database server has 6 IBM Power6 processors at 4.7GHz each with 62 GB of RAM and 1,500 GB of disk storage on a RAID5 configuration. To ensure high availability, our servers are mirrored at data centers in different geographical locations. In addition to hosting mirDIP, we also host other portals such as I2D and GeneCards.

**NOTE:** just for the review process, we provide login (case sensitive):

Username: GenomeBiology

Password: Review
